# Hemoperitoneum in a Cirrhotic Patient Due to Rupture of Retroperitoneal Varix

**DOI:** 10.1155/2009/240780

**Published:** 2009-04-23

**Authors:** Igor Rafael Sincos, Grace Mulatti, Sheila Mulatti, Ilana Cristina Sincos, Sergio Q. Belczak, Valdir Zamboni

**Affiliations:** ^1^Department of General Surgery of Universitary Hospital and Clinics Hospital, University of São Paulo Medical School (FMUSP), 05403-000 São Paulo, SP, Brazil; ^2^Section of Vascular Surgery, Clinics Hospital, University of São Paulo Medical School (FMUSP), Avienda Dr. Enéas de Carvalho Aguiar 255, 05403-000 São Paulo, SP, Brazil

## Abstract

The rupture of retroperitoneal varices is a rare and catastrophic complication of portal hypertension. We describe a case of this nature, the first in Brazilian medical literature, and also reviewing all previous 34 cases. We systematically analyzed all therapeutic approach and propose a management algorithm for diagnosis and treatment of this lethal condition. The majority of the patients presented with abdominal pain, distention and hypotension, and developed hemorrhagic shock. Rupture of retroperitoneal varices can be properly managed if an early diagnosis is made and surgery is performed promptly, which is the only effective treatment. Arteriography should be used when the suspicion is of rupture of hepatocellular carcinoma.

## 1. Introduction

The first case of rupture of retroperitoneal varices, a rare and catastrophic complication of portal hypertension, has been reported in 1958 [1]. According to our research over Medline and PubMed, to date, only 34 similar cases have been described with high mortality rates even nowadays [1–38]. We used hemoperitoneum and varices as keywords for search. The aim of this article is to describe a case of this nature, the first in Brazilian medical literature, and also reviewing all previous cases. We systematically analyzed all therapeutic approaches and propose a management algorithm for diagnosis and treatment of this lethal condition. Rupture of retroperitoneal varices can be properly managed if an early diagnosis is made and surgery is performed promptly.

## 2. Case Report

A 51-year-old woman entered the Emergency Department of Universitary Hospital of the University of Sao Paulo (USP) in March 2006 presenting with abdominal pain for two days, associated with nausea and vomits. She also reported abdominal distention for the last fourteen days.

Her past medical history showed a chronic abuse of alcohol leading to liver cirrhosis associated to Hepatitis B. She had been under medical follow-up with a clinician from 2001 to 2004, when she abandoned medical care.

Her physical examination was remarkable for an ill-appearing, pale, jaundiced, and dyspneic patient. She had a heart rate of 100 beats/min and a systolic blood pressure of 95 and diastolic of 65 mm Hg. Abdominal examination revealed diminished bowel sounds, a slight distention, and diffuse tenderness during palpation, with no guarding. Admission laboratory values showed hemoglobin level of 6.4 g/dL. MELD score of 24, Child-Pugh grade C.

An endoscopy was carried out, which showed a healed distal esophageal ulcer, a hiatal hernia, and erosive gastritis of the body and antrum. There were no signs of esophageal varices.

A few hours after entering the Emergency Department she developed severe hypotension of 70/40 mm Hg, Glasgow coma scale 14, tachycardia of 125 beats/min. She underwent volume resuscitation with no sustained response. Treatment for Spontaneous Bacterial Peritonitis was initiated with Ceftriaxone. Additional treatment with norepinephrine was started as she remained hypotensive even after the continuous infusion of volume. She was then transferred to the intensive care unit. She had progressive hemodynamic instability, abdominal distention, and altered mental status, requiring endotracheal intubation.

At this time a surgeon was requested to examine the patient. A paracentesis was carried out; the peritoneal fluid was hemorrhagic with a hematocrit of 12%. Laboratory values at this moment were hemoglobin level of 3.8 and INR of 7.29. She was then transfused with packed RBCs and plasma. Reaching hemodynamic stability she underwent an exploratory laparotomy.

**Figure 1:**
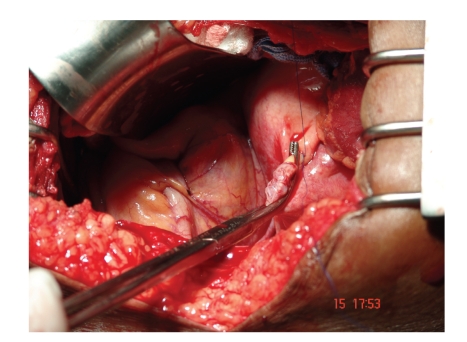
Direct ligation of the vessel in retroperitoneum.

About 5 L of blood were evacuated from the peritoneal cavity. A ruptured retroperitoneal varix was found to be the cause of bleeding, next to the mesenteric root. Direct ligation of the vessel led the bleeding to stop (Figure 1). The liver was cirrhotic; an exuberant collateral circulation was seen on the retroperitoneum and on the abdominal wall, with canalization of the Umbilical vein, with an 8 mm diameter. The Retzius vein was identified in both paracolic gutters, with a lot of collaterals. A liver biopsy was made. Later histopathological evaluation revealed steatohepatitis grade IV (alcoholic and nonalcoholic).

Returning to the intensive care unit the patient was massively transfused with packed RBCs and plasma for anemia and coagulopathy. She continued to be hemodynamically unstable associated with renal and hepatic failure. On the sixth posoperatory she died of multiple organ dysfunction syndrome.

## 3. Discussion

Trauma and nonmalignant gynecological conditions account for more than 90% of intraperitoneal hemorrhages [2]. The main cause of hemoperitoneum in women is the rupture of an ectopic pregnancy; in men, the major cause is the posttraumatic rupture of the liver or spleen [2]. The vascular causes are also relevant, as the Aorta Aneurysm, rupture of viscera, and hemorrhagic pancreatitis. Inflammatory and hematological disorders rarely manifest as hemoperitoneum.

In cirrhotic patients with ascites the intraperitoneal bleeding occurs most of the times due to structural lesions such as hepatocellular carcinoma or ovary cancer and rupture of intraperitoneal varices [3].

The intraperitoneal varices rupture is a rare event, whose incidence unknown, and it is related to severe portal hypertension. We believe that the real incidence of this pathology is much superior than the 34 cases described in literature due to misdiagnosis. It also appears in patients with terminal liver disease, mostly in a fulminate way.

Portal hypertension leads to the development of portosystemic shunts in well-defined anatomic sites. The most acknowledged sites include the gastroesophageal veins connecting the azigohemiazigos system, the hemorrhoidary veins from the inferior mesenteric vein, communicating with the tributaries of the Internal Iliac Vein and the Umbilical and periumbilical veins draining to the left Portal Vein and to the epigastric veins of the anterior abdominal wall. The recanalization of the Umbilical vein is known as Cruveillier-Baumgarten Syndrome [1, 4]. There are also shunts of the Retzius veins connecting the Colic veins with the lumbar and the lower intercostals veins; pancreatic veins connecting the splenic vein and the left renal vein; numerous venous canals communicating the liver with the diaphragm (Sappey veins) [1].

There are 34 cases of intraperitoneal bleeding due to rupture of varices described in literature, as shown in Table 1. Including our patient, 27 were men and 8 were women. The age onset was 21 to 76 years old (average of 48.8). Only one case reported in literature was of a noncirrhotic patient. Table 1 summarizes the presentation, diagnosis, treatment, and results of the cases described in literature.

The majority of the patients presented with abdominal pain, distention and hypotension, and developed hemorrhagic shock. The diagnosis was established by paracentesis, angiography, ultrasound Doppler, and tomography. Even so, the diagnosis was confirmed only by laparotomy.

The hemoperitoneum diagnosis is confirmed by paracentesis when the Ht > 5%. The paracentesis was important in the diagnosis and surgical indication is most of the cases. Only two cases out of ten who survived did not undergo the paracentesis [5, 6]. In one of them, the diagnosis was suggested via tomography and confirmed at laparotomy [5].

The angiography was used as an attempt to achieve the diagnosis in 6 cases of hemoperitoneum by varices inside the abdomen [7–12]. The source of the bleeding was identified only in one patient. However, this patient past away probably because of recurrent bleeding [8]. In one case the angiogram may have anticipated the recurrent bleeding and finally his death [9].

The only effective treatment was surgery. None of the 7 patients treated in a nonchirurgical basis survived. Twenty eight were operated, twelve survived. The global mortality rate was 65.7%. And for the patients submitted to surgery it was of 57.1%. The causes leading of death were uncontrollable or recurrent bleeding, liver failure, kidney failure, heart failure, and aspiration of blood from ruptured esophageal varices.

Out of 28 cases that underwent surgery, in twenty six the ligation of the bleeding vessels was successful, and eleven survived the after surgery period. In two cases who underwent surgery, the ligation was not possible [12, 13], and the patient died of bleeding, even after the use of a portocaval shunt as an attemptive to relieve the portal hypertension [12]. Only one patient survived after a porto-systemic shunt [14].

The management of the bleeding from intra-abdominal varices is difficult since there are no randomized trials due to the rareness of this situation [15]. However, this condition seems to be underestimated, probably because most of the patients present tense ascitis and hepatic disease in a terminal state leading rapidly to death [2].

The patients' survival rate seems to be related to three important facts: the patient's functional hepatic reserve, the importance of the hemorrhagic shock in its presentation, and the early operative intervention and control of the bleeding source [3, 16].

**Figure 2:**
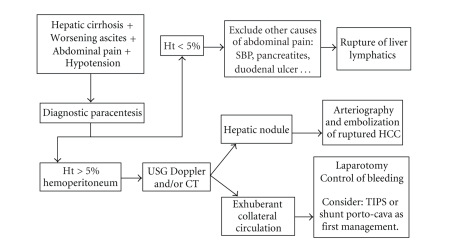
Fluxogram for diagnosis and treatment of hemoperitoneum in cirrhotic patients. SBP: Secondary bacterial peritonitis; Ht: Hematocrit; US: Ultrassonography; CT: Computerized abdominal tomography; HCC: Hepatocellular carcinoma; TIPSS: Transjugular intrahepatic portosystemic shunt.

The first challenge in the management of these cases is in the differential diagnosis of acute hemorrhagic abdomen in a cirrhotic patient. We suggest a flowchart based on the analysis of all the published cases related to intraperitoneal varices and a review of the articles related to the other causes of hemoperitoneum in cirrhotic patients (Figure 2).

The paracentesis with Ht over 5% is a precise indicator of intra-abdominal bleeding that can dimish the risk of unnecessary laparotomies [2]. It may be repeated in another site to exclude a punction accident and has rarely been to relate to hemorrhagic complications [16].

Once the bleeding in a cirrhotic patient was identified, the diagnostic orientation is made on differing HCC rupture, bleeding intra-abdominal varices, vascular causes such as aorta's aneurism, and gynecological causes.

We suggest checking the dosage level of HCG in women, followed by Abdominal Duplex Scan in both sexes as the first diagnostic step. Duplex scan can provide information on the Aorta and its branches, the abdominal collateral circulation, the patency of the Portal Vein, hepatic nodules and tumors, and the ovaries.

Computerized tomographic scanning was suggested by Bataille et al. [15] and Goldstein et al. [5] as the first diagnostic approach for excluding rupture of hepatocellular carcinoma and all other causes. It is, though, more expensive than the Duplex Scan, and it is not always available in all emergency services. On the other hand, it provides more detailed information on other acute hemorrhagic abdomen.

Arteriography has proved to be an inefficient investigation for diagnosis and treatment of retroperitoneal bleeding varices. It postpones the surgical treatment [14], the only one that has been effective in these cases. Thus, it is very important to notice that arteriography has a fundamental role in the treatment of the rupture of hepatocellular carcinoma, which is the main differential diagnosis as mentioned before.

The fundamental treatment of variceal bleeding is the ligation of the vessel. Nevertheless, the Surgical Portosystemic Shunt or the Transjugular Intrahepatic Portosystemic Shunt (TIPSS) must be considered for selected patients.

In the operation room, the decision of performing a Portosystemic shunt must take into consideration the patient's clinical condition and the time needed to perform the shunt. In unstable patients and with little hepatic functional reserve we strongly suggest not increasing the surgical time. However, multiple bleeding varices or the possibility of a new bleed should be analyzed in order to decide if the shunt must be performed.

TIPSS was not performed in any of the reported cases. Nevertheless, the way we understand its use for treatment of gastroesophageal varices can help us in treating variceal bleeding from the retroperitoneum. Therefore TIPSS can be used mainly for the patient's postoperatory when there is suspicion of a new bleeding, or for diminishing the portal tension in selected patients serving as a bridge for liver transplantation. Before deciding if the TIPSS will be performed, one must be aware of its contraindications and complications as bleeding, perforation of the liver capsule, and encephalopathy, among others.

## 4. Conclusion

Bleeding intraperitoneal varix is a rare complication of portal hypertension, but carries a high mortality rate. Nonetheless, the physician must know this condition, as the clinical suspicion is the only way of establishing an early diagnosis and indicating surgery at once, which is the only effective treatment.

We suggest a flowchart to optimize the treatment of the acute hemorrhagic abdomen in the cirrhotic patient. Paracentesis followed by ultrassonography with Duplex Scan or Computerized Tomography seems to be the most important procedure for establishing the correct diagnosis of abdominal pain, distention, and shock. Thereafter, surgery must be performed as soon as possible in case of ruptured varices. Arteriography should be used when the suspicion is of rupture of hepatocellular carcinoma.

## Figures and Tables

**Table 1:** Summary of Presentation, Management and Results of all 35 cases.

Patients *n*: 35 (%)
*Signs and symptoms*	
	
Hypotension or shock	24 (68.6%)
Abdominal pain	23 (65.7%)
Abdominal distention	15 (42.8%)

*Diagnosis*	
	
Paracentesis	22 (62.8%)
Arteriography	6 (17.1%)

Source of bleeding*	
	
Umbilical veins	7 (20%)
Retzius veins	6 (17.1%)
Retroperitoneal varices	5 (14.3%)
Other intraperitoneal sources	19 (54.2%)

*Treatment* ^*#*^	
	
Variceal or vein ligation	27 (77.1%)
Clinical management	6 (17.1%)
Portocaval shunt	3 (8.6%)
Arteriography (embolization)	1 (2.8%)

*Outcome*	
	
Death	23 (65,7%)
Survival	12 (34,3%)

*Some patients had more than one source of bleeding. ^*#*^Some patients were submitted to more than one treatment.
